# Supplementary material for: Gel Synthesis of Hexaferrites Pb1−xLaxFe12−xZnxO19 and Properties of Multiferroic Composite Ceramics PZT–Pb1−xLaxFe12−xZnxO19
Source: Nanomaterials (Basel). 2020 Aug 19;10(9):1630. doi: 10.3390/nano10091630 (PMC7559289; doi:10.3390/nano10091630)
Supplement: Supplementary file 1 [file nanomaterials-10-01630-s001.pdf]

Gel synthesis of hexaferrites $Pb_{1-x}La_xFe_{12-x}Zn_xO_{19}$ and properties of multiferroic composite ceramics PZT - $Pb_{1-x}La_xFe_{12-x}Zn_xO_{19}$

Inna V. Lisnevskaya and Inga A. Aleksandrova

Table 1. Dielectric and magnetic properties of $Pb_{1-x}La_xFe_{12-x}Zn_xO_{19}$ ceramics.

x	ε/ε₀	tanδ	R, Ohm·cm	Hc, kOe	Ms, emu/g	Mr, emu/g
0	45	0.52	$3 \cdot 10^9$	3.0	48.2	23.0
0.1	40	0.32	$5 \cdot 10^9$	2.7	50.1	23.2
0.2	45	0.67	$2 \cdot 10^9$	3.2	53.1	26.1
0.3	65	0.87	$1 \cdot 10^8$	3.0	50.2	26.0
0.5	35	0.56	$6 \cdot 10^8$	2.1	50.1	23.8

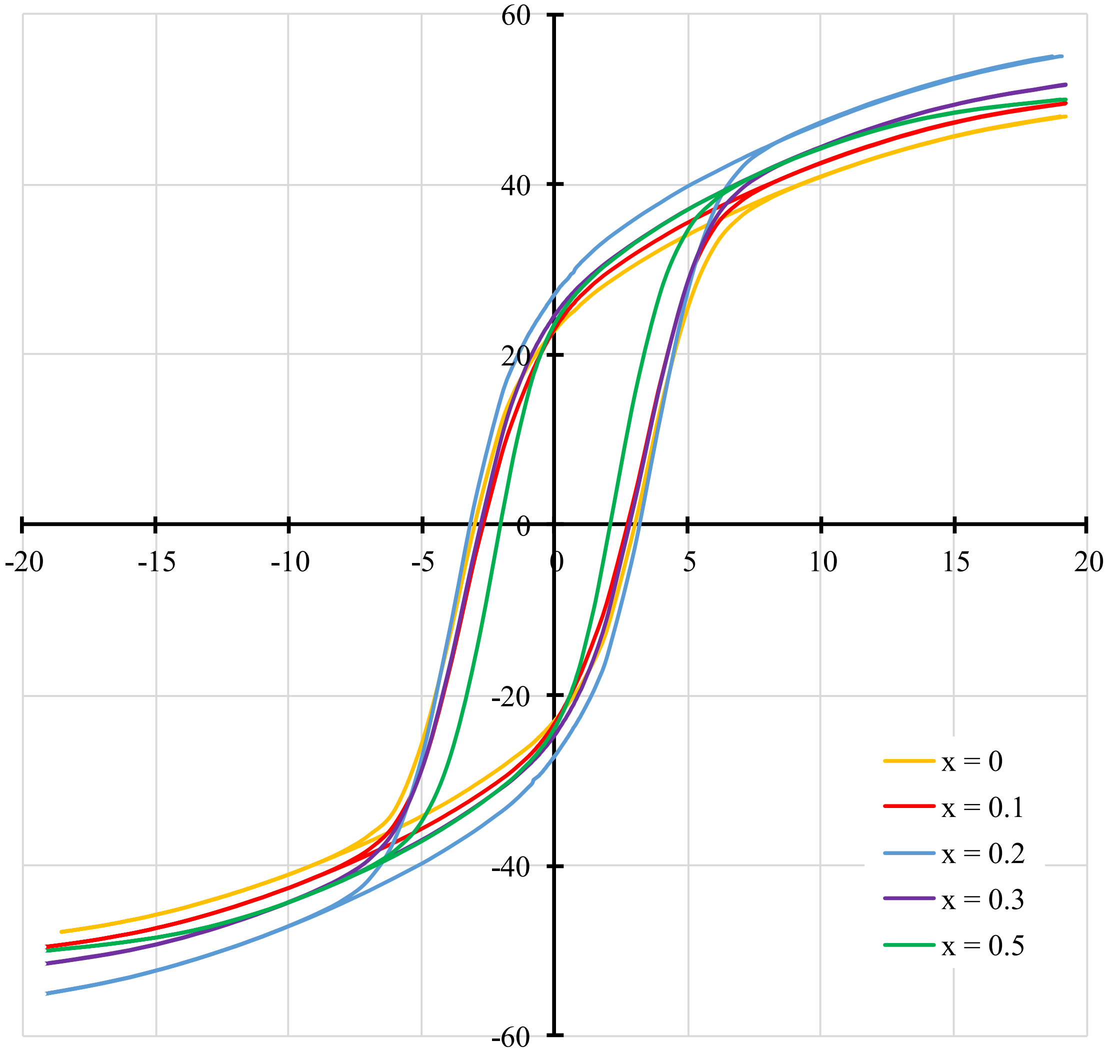

Figure S1. Magnetic hysteresis loops for $Pb_{1-x}La_xFe_{12-x}Zn_xO_{19}$ hexaferrite ceramics.

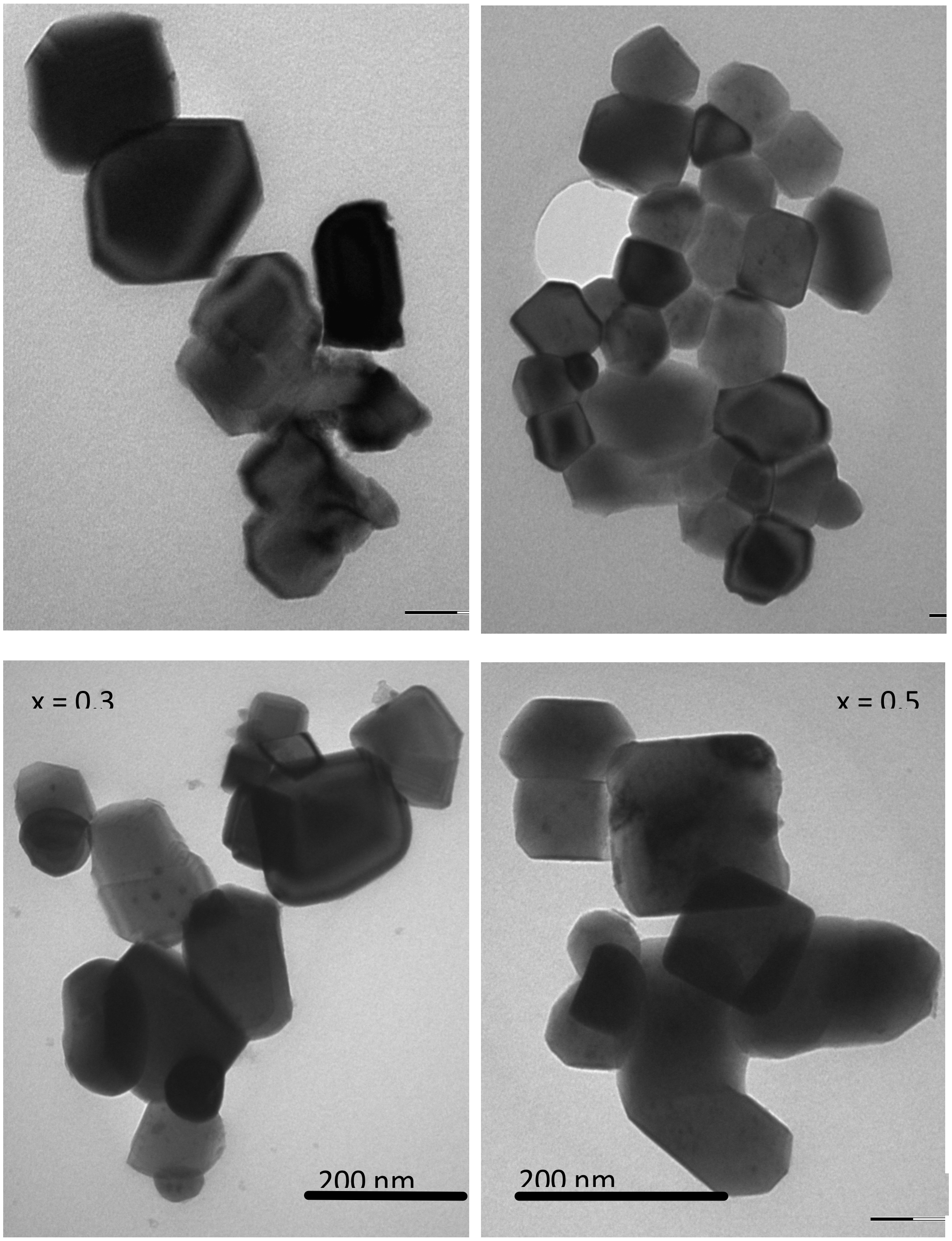

Figure 2. TEM images of $Pb_{1-x}La_xFe_{12-x}Zn_xO_{19}$ nanoparticles synthesized by gel processing.

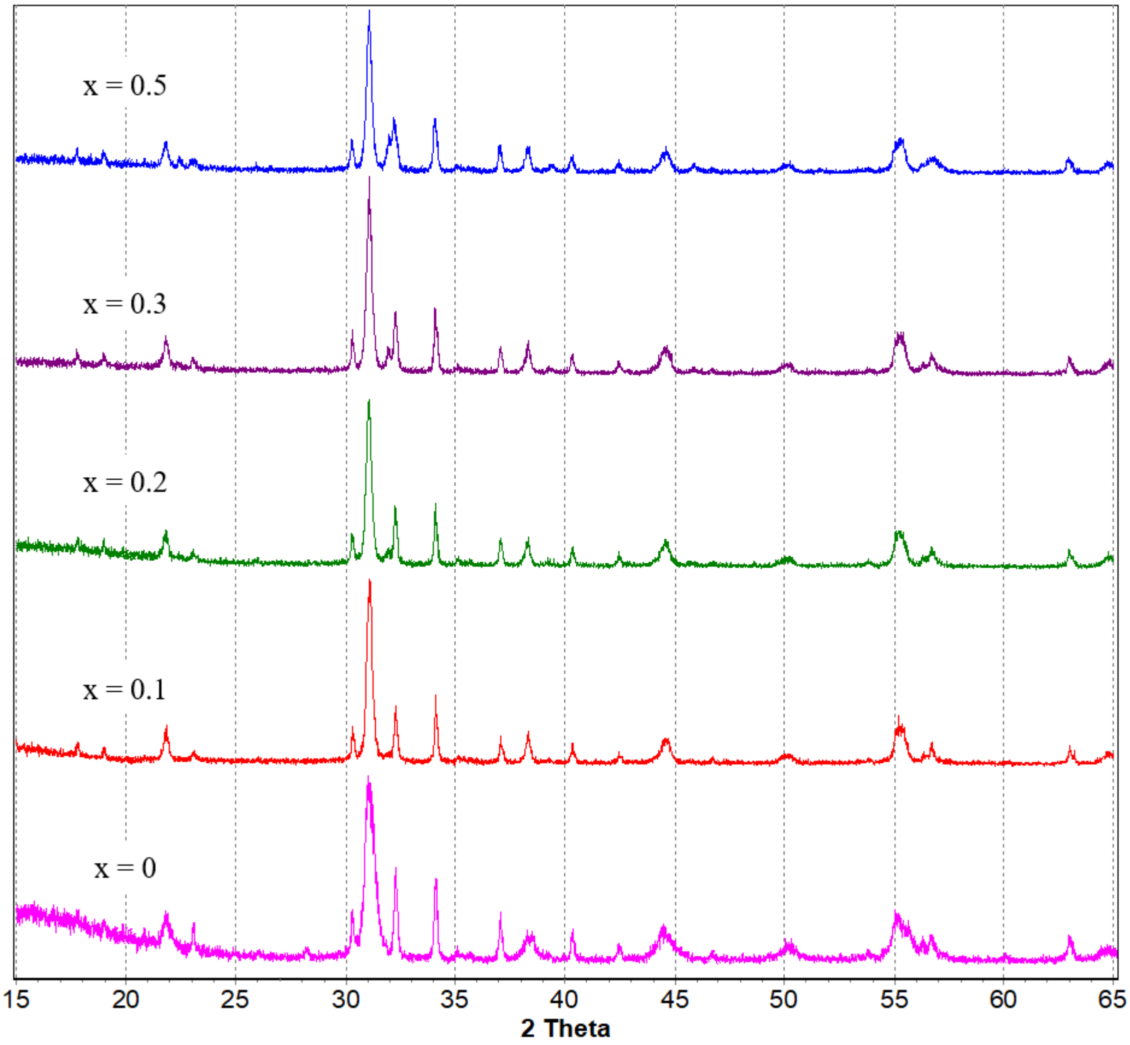

Figure 3. Powder X-ray diffraction patterns of composite ceramics.50 wt. % PZTNB-1 + 50 wt. % $Pb_{1-x}La_xFe_{12-x}Zn_xO_{19}$.
